# Optimizing spatial normalization of multisubject inner ear MRI: comparison of different geometry-preserving co-registration approaches

**DOI:** 10.1038/s41598-025-90842-2

**Published:** 2025-02-21

**Authors:** Johannes Gerb, Valerie Kirsch, Emilie Kierig, Thomas Brandt, Marianne Dieterich, Rainer Boegle

**Affiliations:** 1 German Center for Vertigo and Balance Disorders (DSGZ), LMU University Hospital, Ludwig-Maximilians-University (LMU) Munich, Marchioninistraße 15, 81377 Munich, Germany; 2 Graduate School of Systemic Neuroscience, LMU Munich, Munich, Germany; 3 Department of Neurology, LMU University Hospital, LMU Munich, Munich, Germany; 4 Munich Cluster for Systems Neurology (SyNergy), Munich, Germany

**Keywords:** Endolymphatic hydrops, Vertigo, Peripheral vestibular system, Inner ear, Spatial normalization, Atlas, Sensory systems, Neurology, Inner ear, Translational research

## Abstract

Spatial normalization of multisubject inner ear imaging data is challenging, due to both substantial intraindividual differences and the small size of the organ compared to other intracranial structures. Automatic whole brain co-registration to standard space can only roughly co-align the peripheral vestibular endorgan, and complemental manual registration is highly time-consuming. Here, we compared the accuracy of four geometry-maintaining co-registration methods (one semi-manual method and three automatic methods). High-resolution structural T2-MRI of 153 inner ears from patients and healthy participants were co-registered to an inner-ear atlas. The semi-manual method used a three-point landmark-based approach (3P), two automatic methods were based on unassisted standard algorithms (Advanced Normalization Tools (ANTs), Elastix (EL)), while the fourth automatic method utilized a volumetrically dilated, atlas-based mask (thick inner ear, TIE) for probabilistic inner ear masking. Registration accuracy was evaluated by neurotologists blinded to the respective registration paradigm, and the resulting median volumes were quantified using colocalization analyses. The mask-aided automatic approach showed the best ratings, followed by the semi-manual three-point landmark-based registration (mean ratings (lower: better) TIE 2.21 ± 1.15; 3P 2.58 ± 0.61; EL 3.42 ± 1.06; ANTs 3.49 ± 1.26). The semi-manual method had the lowest rate of insufficient registrations, followed by TIE (3P: 3.70%; TIE: 8.28%; EL: 22.66%; ANTs: 27.02%). TIE showed the highest colocalization metrics with the atlas. Only TIE and 3P allowed for sufficient semicircular canal visualization in method-wise average volumes. Overall, geometry-preserving spatial normalization of multisubject inner ear imaging data is possible and could allow groupwise examinations of the bony labyrinth or temporal bone morphology in the future.

## Introduction

Inner ear anatomy is known to be highly variable^1,2^. Already in healthy participants, diameter and orientation of the semicircular canals (SCC) vary widely, sometimes even intraindividually^1,3^. Disorders can involve SCC, the vestibular aqueduct (VA) or cochlea^4,5^. No clear correlation between this anatomical variance of the bony labyrinth and peripheral vestibular function has been reported thus far, although an influence of temporal bone anatomy on caloric nystagmus intensity has been postulated^6^, and a relevant impact on, e.g., therapeutical effectiveness of positioning maneuvers in benign paroxysmal positional vertigo (BPPV) seems probable^7^. Systematic analysis, however, has been hindered by the lack of standardization in inner ear imaging data analysis.

Multiple approaches of automatic inner ear segmentation, based on MRI^8,9^ or CT scans^10^, have been proposed. Typically, they show good to very good segmentation performance (as evaluated by, e.g., the Sørensen–Dice coefficient^11^) and work with little to no user input. Importantly, these methods only work on individual datasets, and do not allow for groupwise investigations. Co-registration of multisubject or multisubject/multimodal data to a common space, i.e., a shared coordinate system, is usually not performed. This need for standardized spatial coordinates, which are crucial to allow for groupwise image-based analysis, has been recently recognized by Copson et al.^12^. The potential implications for vestibular research become even more evident when considering recent advances in Menière’s disease (MD) research where petrous bone anatomy has been shown to correlate with disease progression^13^. Furthermore, while delayed inner ear imaging for endolymphatic hydrops (ELH) quantification has been used for many years^14,15^, only recently has the focus shifted to extra-labyrinthine structures like the endolymphatic duct^16–18^ or the endolymphatic sac^19^. Omitting petrous bone tissue from MRI analysis pipelines (as is commonly done in automatic segmentation) might subsequently miss relevant information which could aid in differential diagnosis^16,20^.

Multiple approaches aiming at fast and accurate rigid co-registration of inner ear imaging datasets have been proposed^21,22^, often with a focus on cochlear structures in order to facilitate cochlear implant (CI) surgery^23,24^. However, no consensus between different laboratories exists on the optimal preprocessing procedures.

In other fields of neuroimaging, the definition of common imaging coordinate systems (e.g., by the Montreal Neurological Institute (MNI)^25,26^ or in Talairach space^27,28^) have been standard for decades and paved the way for exact colocalization of anatomical structures with, e.g., functional imaging data or cytoarchitectonic atlases, leading to increased understanding of physiological and disease-related brain function^29,30^. This step enables group analyses detached from individual data variability and allowed the development of techniques such as voxel-based morphometry (VBM^31^) and the creation of generalized human connectomes, e.g., in the Human Connectome Project (HCP^32^). For all of these data analysis pipelines with exact colocalization are crucial^33,34^.

**Fig. 1:**
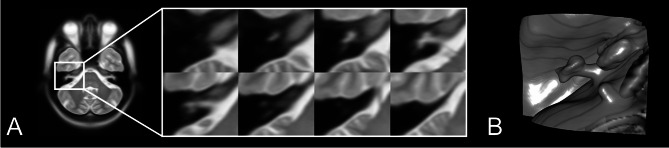
Visualization of the inner ear currently available in the MNI152^20^, based on whole-brain coregistration from 152 healthy adults. While the resulting atlas has been used in countless neuroimaging studies as a standard coordinate system, the inner ear anatomy is not depicted in a sufficient resolution. (**A**) Overview of the MNI high resolution T2 atlas and zoom into the inner ear region. (**B**) 3D-rendering of the inner-ear anatomy available in the MNI152.

Recently, a freely-available atlas for neurotological research has been introduced by Ahmadi et al.^35^, which can be used as a standard coordinate system. With the end-goal of developing VBM-similar techniques for the field of neurotology, reliable methods of coregistration of multisubject data are required. Given the anatomical variance of the structures involved, whole brain coregistration, however, does not result in usable shared coordinates for detailed inner ear anatomy (Fig. 1). Furthermore, automatic registration tools for whole-brain images or cropped petrous bone images methodically often result in misaligned inner ear structures for a simple reason. Since automatic coregistration algorithms work by (1) randomly selecting a given number of voxels, (2) calculating a metric for image similarity (e.g., Dice coefficient), and then (3) performing 2D or 3D transformation operations to optimize this metric until a predefined cutoff value for convergence is reached, small structures tend to be underrepresented. Inherently, voxels from larger structures in a given dataset will statistically be selected more commonly, leading to better alignment of large structures, but potential misalignment of smaller structures. This becomes even more problematic if anatomical structures show large variance, as the inner ear does. These challenges can be mitigated by introducing masks of relevant image areas. This approach has been previously demonstrated for brainstem coregistration^36^.

To fully understand potential disease-specific differences in peripheral vestibular disorders outside of overall inner ear anatomy variance, precise, inner ear–centered coregistration to a common space could therefore enable new analysis methods. Additionally, spatial normalization can aid in existing analyses, e.g., in semiquantitative ELH grading, by allowing exact slice selection as specified in the respective grading system (e.g., midmodiolar level for most grading conventions^37–39^). Importantly, the registration algorithm needs to be adapted to the specific task since all methods expect rigid paradigms (which only allow translation and rotation around the x-, y- and z-axis, but forbid other image alteration) inherently alter the dataset’s geometry. Paradigms with higher degrees of freedom (e.g., allowing for shearing or scaling) or non-linear deformations might result in higher coregistration overlap scores, but ultimately omit geometrical information and render measurements of geometrical petrous bone anatomical features impossible.

In this methodological investigation, we will therefore compare the accuracy on pre-cropped inner ear MRI T2-dataset coregistration from two automatic registration tools with a mask-aided automatic registration and a simple manual registration using anatomical landmarks. The inner ear datasets stem from participants who underwent MRI for diagnosis or exclusion of ELH and the data has been analyzed in previous studies^15,40,41^. Here, those datasets are exclusively used for a methodological investigation in the coregistration performance of T2 high-resolution inner ear MRI. While our primary goal was to determine which method results in the best alignment, our secondary goal was to show how even subpar inner ear registration into a common space still constitutes a significant step towards better data comparability and should be considered when performing inner ear imaging research.

## Methods

### Setting and institutional review board approval

All data was acquired at the interdisciplinary German Center for Vertigo and Balance Disorders (DSGZ) and the Department of Neurology of LMU University Hospital, Munich, between 2016 and 2019. The data protection clearance and Institutional Review Board of the Ludwig-Maximilians-University, Munich, Germany approved the study (no. 641-15). All participants provided informed oral and written consent before inclusion in the study. The study was conducted in accordance with the Declaration of Helsinki and its later amendments.

### Study population

Inner ear datasets from patients with various neurotological disorders who underwent delayed, contrast-enhanced inner ear MRI (iMRI) for exclusion or verification of ELH, were included. Additionally, inner ear datasets from voluntary participants without any history of vestibular disorders were included. These participants underwent contrast MRI for clinical reasons and had previously agreed to perform a second inner ear MRI four hours after the clinical MRI for hydrops evaluation. The inclusion criteria were age between 18 and 85 years and normal kidney function. The exclusion criteria were other neurological or psychiatric disorders, as well as any MR-related contraindications^42^, poor image quality, or missing MR sequences.

### Delayed intravenous gadolinium-enhanced MRI of the inner ear

#### Data acquisition

Four hours after intravenous injection of a standard dose (0.1–0.2 mmol/kg body weight, i.e., 0.1–0.1 mmol/kg body weight) of gadobutrol (Gadovist^®^, Bayer, Leverkusen, Germany), MR imaging (MRI) data were acquired in a whole-body 3 Tesla MRI scanner (Magnetom Skyra, Siemens Healthcare, Erlangen, Germany) with a 20-channel head coil. In this study, only the high-resolution, T2-weighted, spin-echo three-dimensional sampling perfection with application-optimized contrasts by using different flip angle evolutions (3D-SPACE) sequence of the temporal bones was used (TE 133 ms, TR 1000 ms, FA 100°, FOV 192 × 192 mm^2^, 56 slices, base resolution 384, averages 4, acceleration factor of 2 using GRAPPA algorithm, 0.5 mm slice thickness, 0.5 × 0.5 × 0.5 mm^3^ spatial resolution).

#### Data processing

**Fig. 2:**
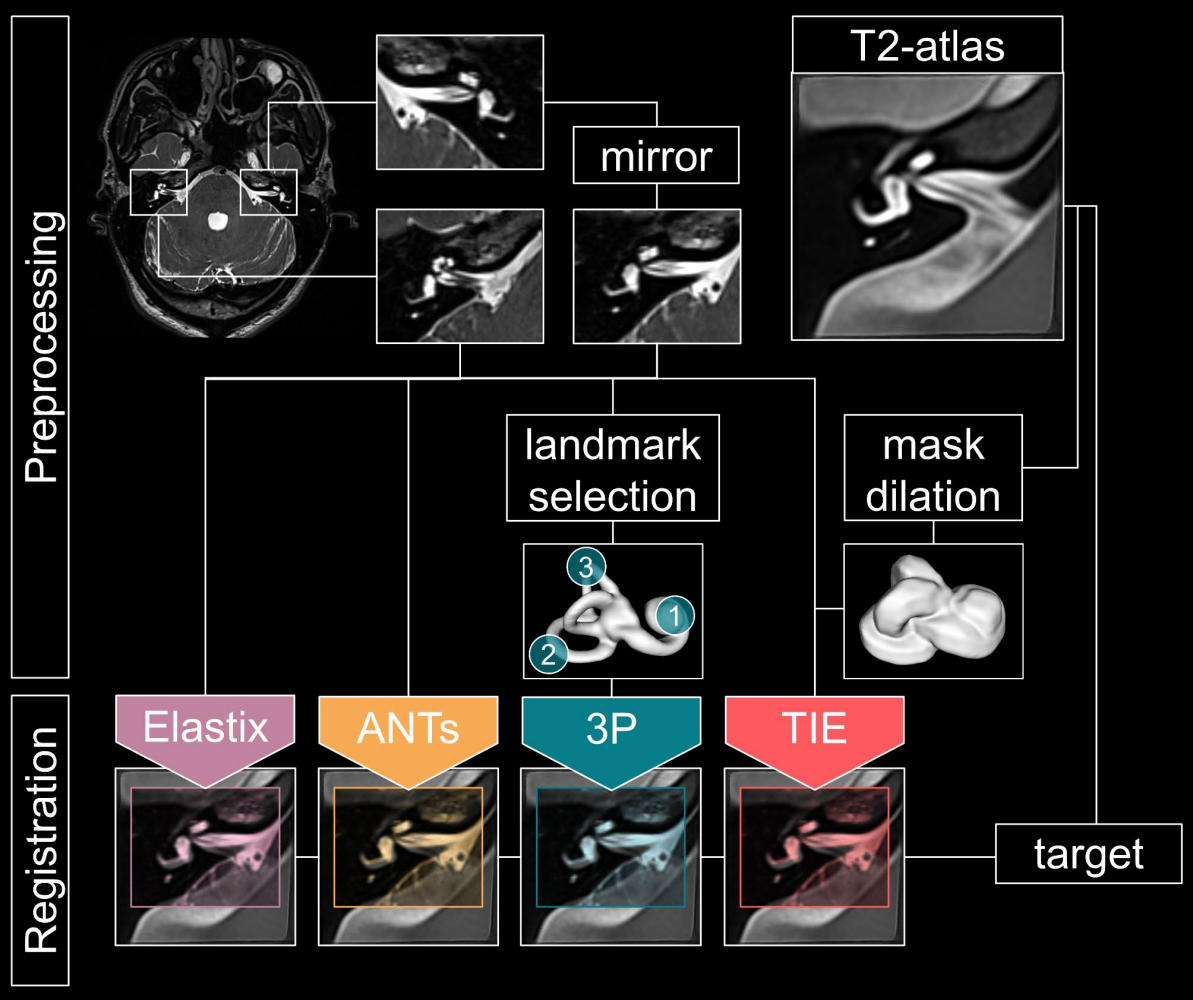
Overview of the preprocessing, registration, and analysis steps for an individual dataset. After selecting both inner ears from the raw T2-SPACE, the left inner ear is mirrored along the sagittal plane to match the orientation of the T2-atlas. For every inner ear crop, two unaided automatic registrations (Elastix, ANTs), one semimanual registration (3P; using three landmarks: (1) apex cochlea, (2) lateral pSCC, (3) superior sSCC), and one mask-aided approach (TIE) were performed (target volume: atlas). While the landmark selection for the 3P-approach was done for every individual dataset, the atlas-derived dilated mask was only created once and used for all subsequent registrations. Registration accuracy was then rated by three experienced neurotologists blinded to the method used. Furthermore, statistical colocalization analysis on the masked registration was performed (not depicted). *ANTs* advanced normalization tools, *3P* three point alignment, *TIE* thick inner ear mask coregistration.

We used ImageJ/FIJI^43^ to select and rescale (two-fold in x-, y- and z-direction, interpolation: bicubic) a rectangular ROI containing both left and right inner ear from the SPACE dataset. Left inner ear datasets were mirrored with respect to the sagittal plane to match the orientation of the right inner ear. All registrations were performed in 3D-Slicer^44^ and the respective implementations of ANTs (Advanced Normalization Tools)^45,46^ and Elastix (EL)^47,48^ were used for registration. Both ANTs and EL are commonly used co-registration toolboxes^49^. For all datasets, the T2 atlas from Ahmadi et al.^35^ was used as a fixed volume (Fig. 2).

For the ANTs-registration, the following parameters were used: Stages: Rigid; initial transform: geometric image center; output interpolation: linear, computation precision: float. For EL, we used the preset “generic (rigid) all”. The TIE approach was performed with the Elastix plug-in using a volumetrically dilated (10px) inner ear mask (mask created in ImageJ/FIJI) defining the region of interest (ROI). The landmark-based approach utilized the “Fiducial Registration”-module to create a transformation matrix which was then hardened using the “Resample image (BRAIN)”—module (interpolation: linear). The landmarks were (1) the outmost (most lateral) part of the posterior SCC, (2) the upmost (most superior) part of the superior SCC under the arcuate eminence and (3) the apex cochleae. All landmarks were selected manually by one of the authors (JG). Landmark selection took roughly 15–20 s per inner ear.

### Subjective grading of coregistration accuracy

All resulting volumes were then given randomized filenames and rated by three of the authors (EK, RB, JG) blinded to the respective registration method used. Method identification was not possible from image characteristics alone. For registration comparison, we used the “Compare Volumes”-module from 3D-Slicer with the atlas as background and the respective registrations as foreground. All raters had multiple years of experience in inner ear imaging and rated each registration on a 6-point Likert scale (1 = best possible alignment, 6 = no alignment/non-convergence). This 6-point grading system is identical to the one being used in German schools and all raters were highly familiar with it. In unclear cases, intermittent half-steps were allowed (e.g., a grade of 2.5 was permitted when the coregistration was subjectively more accurate than other cases graded as 3, but not as accurate as cases graded as 2). Raters were previously instructed to evaluate inner ear geometry rather than, e.g., brainstem coregistration results.

Group averages were created using ImageJ/FIJI^43^ by importing all method-specific volumes as a 4D-hyperstack and then using a slice-wise z-projection for median image intensity. In order to determine image similarities, we used the “Colocalization-Threshold”-plugin in ImageJ (thresholding via Costes auto-threshold^50^, correlation with Spearman’s Rho and Manders split^51^). To remove false-positive colocalization metrics from background noise or, e.g., brainstem tissue, a dilated inner ear mask was used to define the region of interest (ROI).

### Statistical analysis

We used JASP (version 0.18.3, https://jasp-stats.org/*)* to calculate mean method-wise scores per rater and performed ANOVA-testing to compare the average scores per registration method (post-hoc comparisons Bonferroni corrected). For dichotomic variables, Welch’s t-test was used. Inter-rater-agreement was assessed using Spearman’s rho due to the potentially different baseline ratings per rater which would have falsely influenced e.g., Cohen’s kappa^52^. Additionally, Kendall’s tau was calculated in order to be more sensitive regarding tied ranks in the 6-point rating scale.

## Results

107 inner ear datasets from 54 individual patients with various neurotological disorders (mean age 51.3 ± 16.1 years, 29 females) and 46 inner ear datasets from 23 participants without any history of vestibular disorders (mean age 40.0 ± 17.2 years, 13 females) were included, resulting in a total of 153 inner ear datasets. Demographic characteristics of the included inner ears were comparable when divided by patient sex (mean age female inner ears (*n* = 84): 47.6 ± 18.3 years, mean age male inner ears (*n* = 69): 48.3 ± 16.0 years; Welch’s t-test of groupwise mean age not significant: t 0.55, *p* 0.59).

**Fig. 3:**
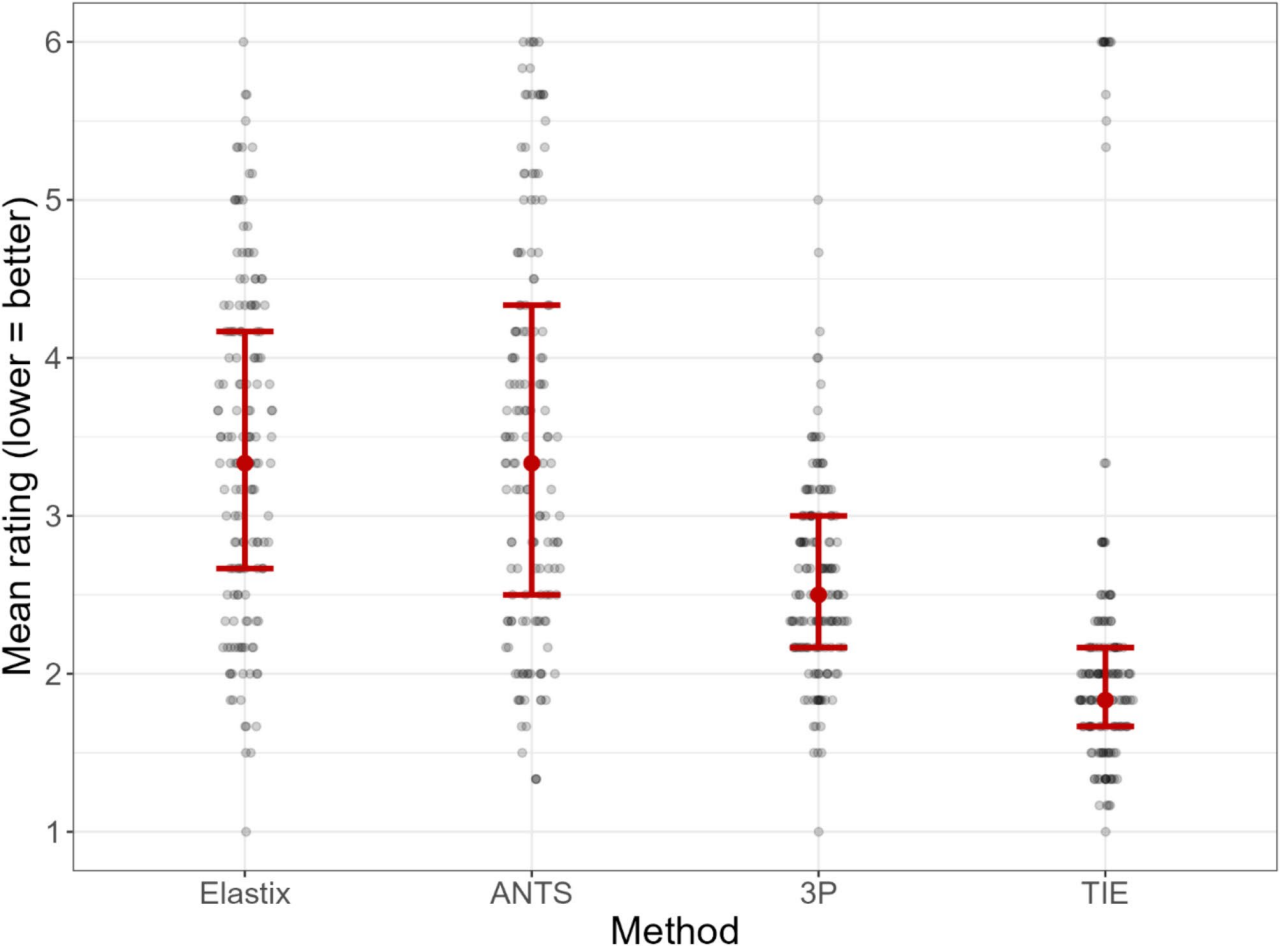
Average ratings of three independent raters, blinded to the method (1 = best possible grade, 6 = worst possible grade; lower average scores equal to better performance). On average, TIE showed the best ratings; however, in some cases, non-convergence occurred. 3P results in slightly less accurate coregistration with almost no cases of non-convergence. Both automatic methods (ANTs, Elastix) showed significantly worse scores. *ANTs* advanced normalization tools, *3P* three point alignment, *TIE* thick inner ear mask coregistration.

The average ratings (with lower scores representing higher subjective accuracy) were 2.21 ± 1.15 for the mask-aided TIE approach, 2.58 ± 0.61 for the semimanual three-point approach, 3.42 ± 1.06 for EL, and 3.49 ± 1.26 for ANTs (Fig. 3). ANOVA testing showed a highly significant effect of registration method on the mean rating (F 3,608 = 55.73, *p* < 0.001), with post-hoc comparison revealing clear differences between the unassisted automatic methods (ANTS, EL) and both the mask-aided and landmark-based approach (ANTs vs. TIE: mean difference 1.28, Cohen’s d 1.22, pBonf < 0.001***; ANTs vs. 3P: mean difference 0.91, Cohen’s d 0.87, pBonf < 0.001***; EL vs. TIE: mean difference 1.21, Cohen’s d 1.16, pBonf < 0.001***; EL vs. 3P: mean difference 0.85, Cohen’s d 0.81, pBonf < 0.001***). Between TIE and 3P, TIE showed significantly better ratings (mean difference 0.37, Cohen’s d 0.35, pBonf 0.01*). No significant difference was found between ANTs and EL (mean difference − 0.07, Cohen’s d − 0.06, pBonf 1.00, all *p* values adjusted for comparing a family of 4).

Due to the subjective nature of the rating system, the mean scores given by the three raters diverged around individual baselines (EK: 2.09 ± 1.36, RB: 3.64 ± 1.31, JG: 3.05 ± 1.27). However, all raters independently rated the TIE approach the best, followed by 3P (mean score TIE: 1.49 ± 1.31 (EK), 2.78 ± 1.20 (RB), 2.31 ± 1.28 (JG); mean score 3P: 1.70 ± 0.72 (EK), 3.35 ± 0.95 (RB), 2.64 ± 0.77 (JG); mean score EL: 2.48 ± 1.32 (EK), 4.27 ± 1.17 (RB), 3.43 ± 1.17 (JG); mean score ANTs: 2.69 ± 1.54 (EK), 4.16 ± 1.31 (RB), 3.64 ± 1.35 (JG); Fig. 3). The overall inter-rater agreement was good, as determined by a correlation analysis using Spearman’s rho (EK/JG: rho 0.65, *p* < 0.001; EK/RB: rho 0.69, *p* < 0.001; RB/JG rho 0.71, *p* < 0.001) and Kendall’s tau (EK/JG: tau 0.58, *p* < 0.001; EK/RB: tau 0.59, *p* < 0.001; RB/JG tau 0.60, *p* < 0.001).

The number of total insufficient registration outcomes (5 and 6 on the 6-point Likert scale, from all three independent raters combined, i.e., from all 612 co-registrations) were *n* = 17 for the semimanual approach, *n* = 38 for TIE, *n* = 104 for EL, and *n* = 124 for ANTs. Raters diverged in their overall number of registrations rated as insufficient (JG *n* = 91, EK *n* = 59, RB *n* = 133). However, both in grouped analysis as well as in rater-wise analysis, the semimanual approach (3P) had the lowest rate of non-convergence, followed by TIE (3P: 3.70%; TIE: 8.28%; EL: 22.66%; ANTs: 27.02%).

No effect of participant sex on the overall registration accuracy was found (Welch’s t-test: n.s.). ANOVA-testing with Bonferroni-corrected post-hoc comparison additionally ruled out method-specific sex effects (TIE mean sex difference: 0.08, pBonf 1.00; 3P mean sex difference: 0.09, pBonf 1.00; EL mean sex difference: 0.13, pBonf 1.00; ANTS mean sex difference: 0.34, pBonf 1.00).

**Fig. 4:**
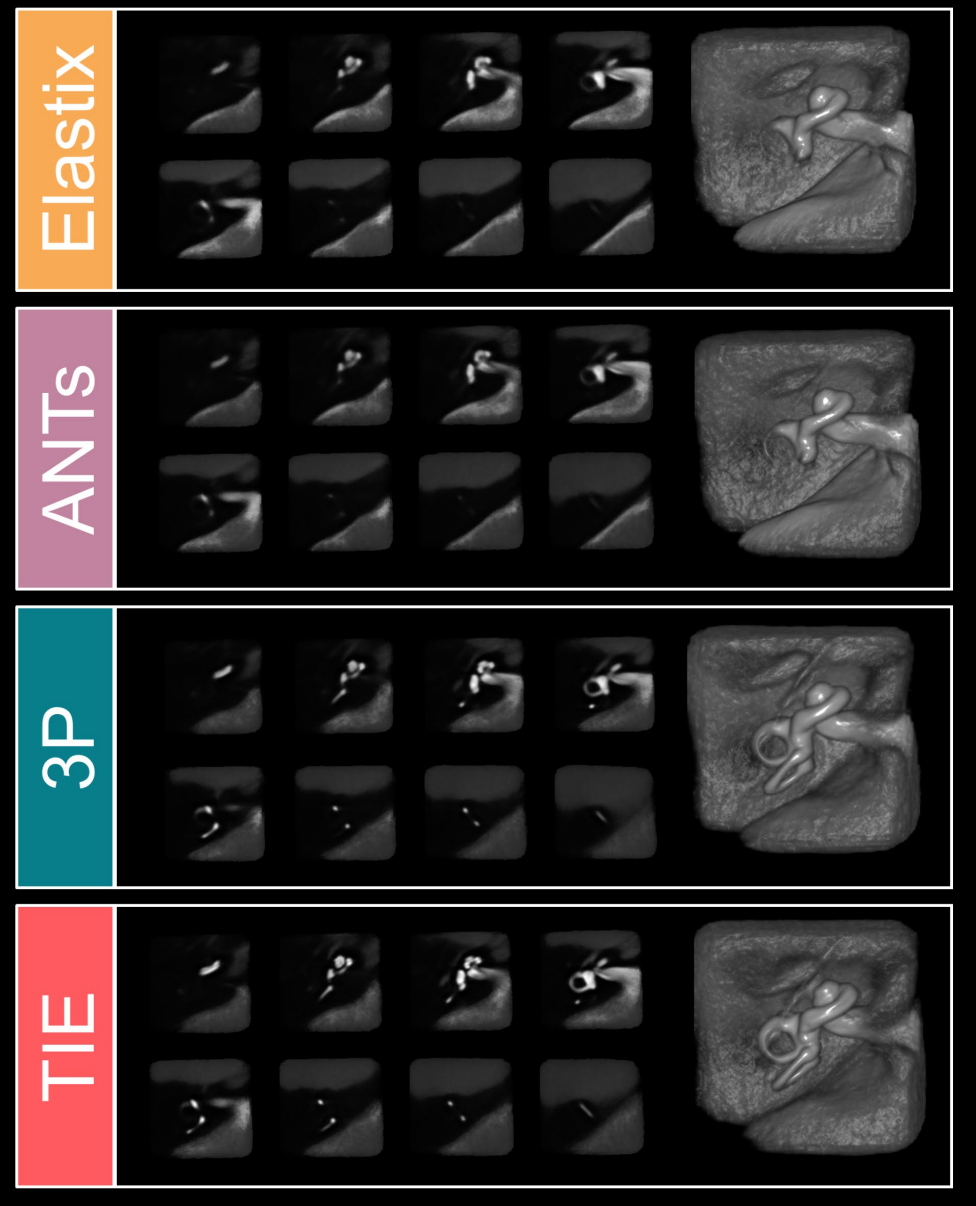
Slicewise comparisons of the 4 examined registration methods and 3D-renderings of the resulting median volumes from 153 human inner ears (from top to bottom: automatic Elastix registration, automatic ANTs registration, semimanual three-point registration (3P), automatic mask-aided TIE-approach). Finer inner ear details such as the semicircular canals are only discernible in the 3P and TIE methods.

Visual analysis of the group-medians showed the highest overlap with the atlas in the TIE-approach and the 3P-method (Fig. 4). The SCCs could not be clearly separated from background noise in group-medians from the unaided registration algorithms (EL, ANTs; Fig. 4).

**Fig. 5:**
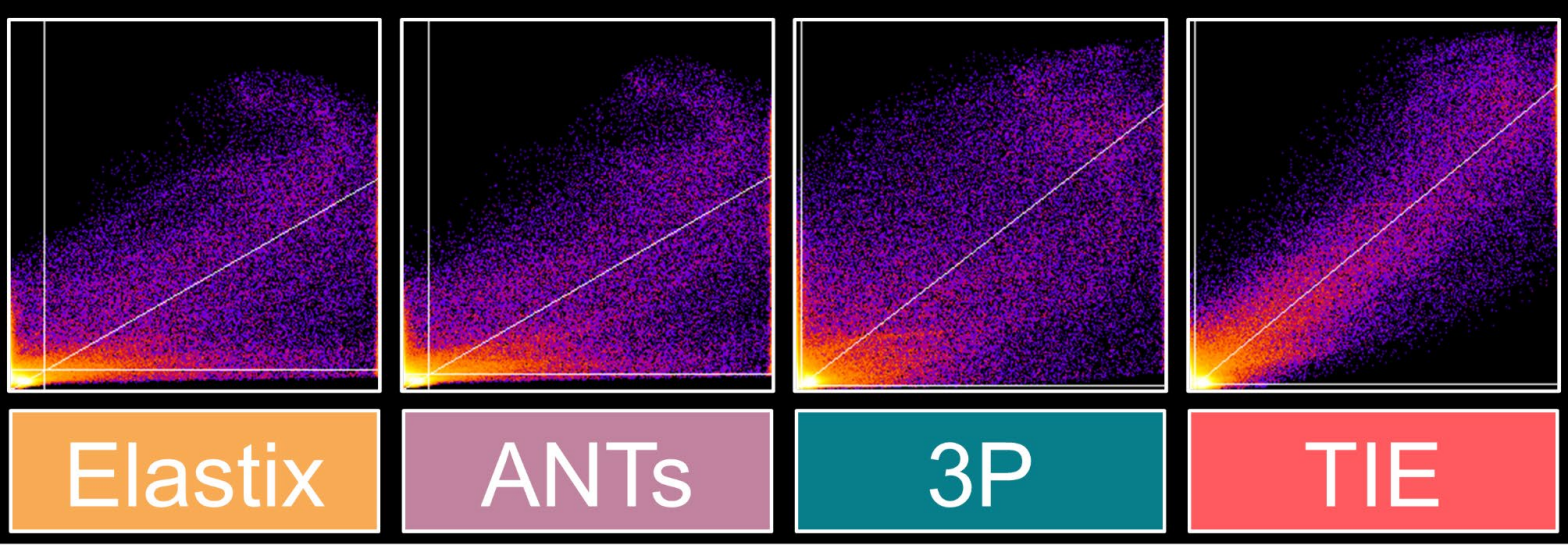
Colocalization scatterplots of each registration method with the target atlas (from left to right: Elastix, ANTs, 3P, TIE). In order to exclude background noise, an inner ear mask was used as an ROI (diagonal line: linear regression fit of colocalizations; horizontal/vertical lines: Costes auto thresholds for channel separation). While exact colocalization (i.e., all data points on the diagonal line) is not possible since the moving image and target image are inherently different, TIE results in the most accurate coregistration, i.e., data points located in close proximity to the diagonal line.

The colocalization analysis between median method-specific volumes and the source atlas showed the highest correlation scores (Spearman’s rho) for the TIE-approach (TIE: 0.931; 3P: 0.780, EL 0.761, ANTs 0.777). The thresholded Mander-Splits (automatic thresholding into two channels) were the highest for the TIE-approach (TIE Channel 1: 0.9796, Channel 2: 0.9530; 3P Channel 1: 0.9926, Channel 2: 0.9170; ANTs Channel 1: 0.8730, Channel 2: 0.7730; EL Channel 1: 0.8092, Channel 2: 0.7546, cp. Fig. 5). A simple linear regression model showed the most ideal fit (i.e., an estimated slope approximating 1) for the TIE approach, followed by 3p (TIE: y = 0.829x + 0.0; 3p: y = 0.777x + 0.1; ANTs: y = 0.581x + 0.2; EL: y = 0.575x − 0.6).

## Discussion

In this methodological investigation, it was shown that despite anatomical variance, automated and fast spatial normalization of multi-subject inner ear imaging datasets to a common space is feasible. From the automatic algorithms, the mask-aided approach using an atlas-based dilated inner ear mask (TIE) significantly outperformed the unaided methods investigated in this study and showed the highest scores overall, both in subjective rating scores and in quantitative colocalization metrics. As a slower alternative, semimanual landmark based inner ear registration using only three fiducials (3P) showed the second-highest ratings, and had the lowest rate of non-convergence. All methods investigated resulted in clearer defined group-wise inner ears than whole-brain coregistrations (Figs. 1 and 4), and would allow basic group-wise atlas-based morphometrical analyses. However, finer inner ear structures such as the SCCs were only discernible in TIE or 3P.

Notably, the subjective rating scores showed no sex differences, indicating an unbiased performance. Especially when considering known sex differences in the vestibular system^53–55^, methodical impartialness is a necessary prerequisite for the analysis of potential sex-specific differences in inner ear anatomy after establishing a stable multi-subject coregistration method.

The comparative evaluation of the current study has some limitations. Firstly, the subjective rating scale to determine the registration performances is intrinsically subjective, despite raters being blinded to the method used. As expected, this resulted in different average scores per rater, with some raters giving better grades overall than others. However, the ranked inter-rater agreement was very high (Spearman’s rank correlation, Kendall’s Tau), and all raters agreed on the superior performance of the TIE approach, followed by 3P, as could be shown in the subjective ratings. While a correlation of subjective and objective coregistration scores would be desirable, conventional colocalization metrics such as the Dice-coefficient suffer from the same methodological drawbacks that make the development of inner-ear centered registration approaches necessary in the first place. Since larger structures tend to be overrepresented in Dice-score calculation, inadequate inner ear registrations can result in higher Dice-scores than adequate ones, if e.g., brainstem or petrous bone tissue coalignment is higher. For this reason, we utilized in the current study colocalization quantification metrics which do not suffer from these limitations. These unbiased metrics (automated thresholding and voxelwise correlation) confirmed the subjective scores, with the TIE-approach and the semimanual method showing the highest performance.

A second potential limitation could be that the landmark selections for all 3P datasets were done by a single author (JG). Here, further research is required to quantify intra-observer and inter-observer variability during landmark selection, and their potential effects on the final registration.

This study did not include major congenital inner ear malformations. All inner ears were from patients and healthy participants with overall normal inner ear anatomy. For such patients, a modified spatial template might be necessary, and the 3P-approach might not be applicable.

All patients included in this study underwent standardized MR imaging with predefined sequences, acquired in a single center on the same scanner unit. However, it should be noted that all datasets were acquired as part of the clinical routine, rather than as part of an elaborate, extensive neuroimaging study: the scanner unit used in this study is a widely available model (Magnetom Skyra, Siemens Healthcare, Erlangen, Germany), and the employed MR sequence (3D-SPACE) constitutes a rather standard inner ear imaging sequence. All employed coregistration methods can be applied to multisite MR data as well, e.g., in multicenter studies, although we did not investigate multisite-harmonization performance in the current study. In principle, however, one major advantage of the semimanual 3P-technique is its generalizability since the three landmarks used in this study should be easily identifiable in high-resolution MR or CT sequences, independent of vendor or sequence characteristics. Future developments could include automatic landmark selection, for example, using deep learning^56^.

On a technical note, we tested the 3D-Slicer implementations of the automatic registration tools which are not as actively developed as the standalone versions. While software updates cannot solve the inherent pitfalls of automatic, unaided inner ear image coregistration, code improvements might still result in notable performance increases in the standalone versions compared to the 3D-Slicer implementations. Importantly, however, the registration inaccuracies observed in the automatic registration tools do not stem from faulty algorithms or their wrongful implementation. The software worked just as intended, correctly optimizing the registration process which inherently (due to the aforementioned anatomical and methodological pitfalls) resulted in misaligned inner ears. This is one of the reasons why no hyper-parameter optimization of the automatic algorithms was performed; while parameter tuning might potentially result in slightly higher accuracy, these inherent pitfalls would not be avoided.

It should be noted that the aim of this study was the analysis of rigid registrations that preserve petrous bone anatomy and geometry. Depending on one’s research question, non-linear registration of a presegmented individual inner ear dataset onto an anatomically labeled inner atlas might suffice, despite inherently altering petrous bone geometry. For this, numerous registration toolboxes with great accuracy are available^49,57^. However, when petrous bone anatomy needs to be analyzed without introducing geometrical modifications, rigid registration is necessary (e.g., with TIE and 3P). As noted earlier, this can only result in approximate colocalization in a multipatient dataset.

## Conclusions

Mask-aided inner ear coregistration, followed by semimanual landmark-based registration, outperformed unaided automatic coregistration methods, while all methods were substantially more accurate than simply a whole-brain coregistration. In general, spatial normalization should be integrated into inner ear imaging data analysis pipelines to aid in research comparability and to lay the foundation for more complex analyses of neurotological datasets. To allow other researchers to implement spatial normalization steps, the TIE method (which proved superior in this analysis) will be made freely available.

## Data Availability

All software used in this study is open source and available for free download. The data that support the findings of this study are available on request from the corresponding author. The data are not publicly available due to privacy or ethical restrictions.
